# Aflatoxin B_1_ Promotes Influenza Replication and Increases Virus Related Lung Damage via Activation of TLR4 Signaling

**DOI:** 10.3389/fimmu.2018.02297

**Published:** 2018-10-04

**Authors:** Yuhang Sun, Jiarui Su, Zixuan Liu, Dandan Liu, Fang Gan, Xingxiang Chen, Kehe Huang

**Affiliations:** ^1^Department of Animal Nutrition and Immunology, College of Veterinary Medicine, Nanjing Agricultural University, Nanjing, China; ^2^Institute of Nutritional and Metabolic Disorders in Domestic Animals and Fowls, Nanjing Agricultural University, Nanjing, China

**Keywords:** aflatoxin B_1_, swine influenza virus, replication, inflammation, lung damage, TLR4, NFκB, TNF-α

## Abstract

Aflatoxin B_1_ (AFB_1_), which alters immune responses to mammals, is one of the most common mycotoxins in feeds and food. Swine influenza virus (SIV) is a major pathogen of both animals and humans. However, there have been few studies about the relationship between AFB_1_ exposure and SIV replication. Here, for the first time, we investigated the involvement of AFB_1_ in SIV replication *in vitro* and *in vivo* and explored the underlying mechanism using multiple cell lines and mouse models. *In vitro* studies demonstrated that low concentrations of AFB_1_ (0.01–0.25 μg/ml) markedly promoted SIV replication as revealed by increased viral titers and matrix protein (M) mRNA and nucleoprotein (NP) levels in MDCK cells, A549 cells and PAMs. *In vivo* studies showed that 10–40 μg/kg of AFB_1_ exacerbated SIV infection in mice as illustrated by significantly higher lung virus titers, viral M mRNA levels, NP levels, lung indexes and more severe lung damage. Further study showed that AFB_1_ upregulated TLR4, but not other TLRs, in SIV-infected PAMs. Moreover, AFB_1_ activated TLR4 signaling as demonstrated by the increases of phosphorylated NFκB p65 and TNF-α release in PAMs and mice. In contrast, TLR4 knockdown or the use of BAY 11-7082, a specific inhibitor of NFκB, blocked the AFB_1_-promoted SIV replication and inflammatory responses in PAMs. Furthermore, a TLR4-specific antagonist, TAK242, and TLR4 knockout both attenuated the AFB_1_-promoted SIV replication, inflammation and lung damage in mice. We therefore conclude that AFB_1_ exposure aggravates SIV replication, inflammation and lung damage by activating TLR4-NFκB signaling.

## Introduction

Swine influenza virus (SIV), a single-stranded negative-sense RNA virus, causes severe systemic effects, resulting in significant economic losses in the animal husbandry industry. SIV also causes human disease and can even give rise to human pandemics, including the pandemic caused by the H1N1/2009 virus (1). Increasing evidence indicates that viral infection is associated with several environmental, nutritional, and immune factors, such as mycotoxin contamination (2, 3), selenium deficiency (4, 5), and macrophage polarization (6). The involvement of these factors may partly explain the differences in morbidity and mortality in infected animals and humans all over the world.

Aflatoxin B_1_ (AFB_1_), which is produced by *Aspergillus flavus*, is one of the most common mycotoxins in contaminated food and plant products from tropical and subtropical areas with high temperature and humidity (7, 8). It is well known that AFB_1_ is harmful to the liver and kidney of mammals and is regarded as a representative orally ingested carcinogen (9, 10). However, increasing evidence indicates that AFB_1_ can also affect immune responses in mammals (11, 12); these evidences show that low doses of AFB_1_ (≤ 0.025 mg/kg) significantly increase the secretion of pro-inflammatory cytokines by T cells and NK cells in rats, but high doses of AFB_1_ (0.4–0.8 mg/kg) markedly decrease macrophage migration and the lymphocyte response to mitogens in pigs. Specifically, some reports propose that mycotoxins can eventually decrease resistance to infectious diseases (13), and aflatoxins are thought to feature prominently in the progression of some viral diseases, such as HIV (3). However, so far, there have been no studies investigating whether influenza virus infection in mammals exposed to AFB_1_ is more severe than infection in unexposed mammals.

Toll-like receptors (TLRs) compose a main family of pattern recognition receptors with a critical role in the activation of the innate immune response (14). To date, there are at least 13 members (TLR1-TLR13) of this family in mammals that recognize specific components of pathogenic microorganisms. TLR4 is a unique receptor for pathogen recognition that was initially found in various cell types, including porcine alveolar macrophages, and in mice. In the past, many studies have focused on TLR4 structure and function. On the one hand, TLR4 activation leads to nuclear factor kappa (NFκB) translocation and the expression of proinflammatory cytokines, including tumor necrosis factor (TNF-α), which is responsible for activating the innate immune system (15). On the other hand, the overexpression or continuous activation of TLR4 leads to excessive inflammatory responses and/or tissue injury in the body (16–19). Interestingly, viruses can evade the host immune response when TLR4 is inhibited, thereby enhancing viral replication, and one study has shown that a TLR4 antagonist can protect mice from lethal influenza infection (20). Nevertheless, few studies are available regarding the role played by TLR4 in AFB_1−_promoted SIV replication.

Thus, given the differences in morbidity and mortality following SIV infection, we hypothesized that AFB_1_ promotes SIV replication. In this study, multiple cell lines and mouse models were established to assess the involvement of AFB_1_ in SIV replication *in vitro* and *in vivo* and to elucidate the underlying mechanism of such involvement.

## Materials and methods

### Ethics statement

This research protocol was approved by the Ethics Committee for Animal Experimentation of Nanjing Agricultural University (approval number: SYXK-SU-2011-0036). All animal care and use procedures were conducted in strict accordance with the Animal Research Committee guidelines of the College of Veterinary Medicine at Nanjing Agricultural University, and all efforts were made to minimize animal suffering and to reduce the number of animals used.

### Reagents

AFB_1_ (1 mg/mL; Sigma-Aldrich, USA), BAY 11-7082 (10 mM; MCE, USA) and TAK-242 (50 mM; ApexBio, USA) were dissolved in dimethyl sulfoxide (DMSO), packaged, and stored frozen at −20°C until use. For *in vitro* studies, the dissolved AFB_1_ was diluted with serum-free medium, and equal concentrations of DMSO were used in the vehicle and in the control solution. For *in vivo* studies, the dissolved AFB_1_ was diluted in fresh sterile endotoxin-free saline daily, and the solution was then injected intraperitoneally (i.p.) at concentrations of 10, 20, and 40 μg/kg b.w.; diluted TAK-242 was also prepared daily and then injected i.p. (3 mg/kg b.w.) 1 h prior to other treatments as previously described (16, 17, 21).

### Cell culture

Madin-Darby canine kidney (MDCK, NBL-2) cells, human lung cancer cells (A549) and porcine alveolar macrophages (PAMs, 3D4/21) that were free of any respiratory or systemic diseases were purchased from the China Institute of Veterinary Drug Control (Beijing, China). MDCK and A549 cells were grown in Dulbecco's modified Eagle's medium (Gibco, USA) containing 10% fetal calf serum (FCS; Gibco, USA) and 1% penicillin-streptomycin (Solarbio, China) at 37°C in 5% CO_2_. PAMs were cultured in Roswell Park Memorial Institute-1640 medium (Gibco, USA) supplemented with 10% FCS, 1% penicillin-streptomycin and 1% nonessential amino acids (Gibco, USA) at 37°C in 5% CO_2_. Cells and serum and culture medium were tested for mycoplasma using MycoTestTM kit (Seebio, China). During viral infection, all cell lines were transferred to serum-free medium supplemented with 1 μg/ml tolylsulfonyl phenylalanyl chloromethyl ketone (TPCK)-treated trypsin (Sigma, USA).

### Cell viability determination by MTT and LDH assays

MDCK cells, A549 cells and PAMs were cultured in 96-well plates for 24 h and were then exposed to various concentrations of AFB_1_ or to 1 μg/ml DMSO for an additional 24 h before being subjected to colorimetric 3-(4,5-dimethylthiazol-2-yl)-2,5-diphenyltetrazolium bromide (MTT) assay. Subsequently, the absorbance was measured at 490 nm with a reference wavelength of 655 nm, and all experiments were performed in triplicate.

Lactate dehydrogenase (LDH) release was also measured using commercially available kits to assess cell viability. Briefly, cells were seeded in 96-well plates and exposed to various concentrations of AFB_1_ or to 1 μg/ml DMSO. After 24 h of incubation, the supernatant was collected for the measurement of LDH release according to the manufacturer's protocol (Jiancheng, China). The absorbance was measured at a wavelength of 450 nm, and all samples were measured in triplicate.

### Apoptosis assay by DAPI staining

4′,6-diamidino-2-phenylindole (DAPI) staining was performed as described previously (22) with a minor modification. Briefly, PAMs were seeded on coverslips (WHB, China) into 12-well culture plates and incubated with AFB_1_ and DMSO for 24 h. Next, the PAMs were washed three times with PBS and fixed with 4% paraformaldehyde for 20 min at 4°C. After three washes, cell nuclei were counterstained with DAPI (Beyotime, China) for 5 min in the dark. Finally, the stained PAMs were washed three times and examined by fluorescence microscopy (Nikon Ti-S, Japan).

### Viral titration by TCID_50_

Influenza virus strain A/swine/Guangxi/18/2011 (H1N1) was kindly provided by Dr. Weiye Chen, Harbin Veterinary Research Institute, Chinese Academy of Agricultural Sciences (Harbin, China). The virus was propagated in MDCK cells, and the supernatant was harvested at 72 h post infection (hpi) to ensure that enough virus was obtained. The viral titers were determined by the 50% tissue culture infectious doses (TCID_50_) in MDCK cells, A549 cells and PAMs. Briefly, the MDCK cells, A549 cells and PAMs were seeded in a 96-well plate (Corning, USA) for 24 h, infected with 10-fold serial dilutions of virus in serum-free medium supplemented with 1 μg/ml TPCK-treated trypsin and then exposed to various concentrations of AFB_1_. The cytopathic effect induced by the virus was observed and recorded after 24 hpi to calculate the virus titers by the method of Reed and Muench. A biosafety level 2 facility was used for all the experiments with the H1N1 virus.

### Animals and study design

Male TLR4 knockout (C57BL/10ScNJNju, TLR4^−/−^) and wild-type (C57BL/10JNju, WT) mice, 6–8 weeks old and weighing 18–20 g, were purchased from Nanjing University (Nanjing, China). TLR4^−/−^ mice do not express functional TLR4 or TLR4 mRNA because of the TLR4 lps-del mutation. All mice were housed in a specific pathogen-free environment (22 ± 2°C) with a 12 h light/dark cycle. Water and food were available *ad libitum* throughout the whole study. All mice were acclimatized for 1 week before the onset of experiments. Body weight changes and illnesses were monitored daily.

For the first randomized trial, WT mice were randomly divided into 6 groups (each group included 3 replicates, with 4 mice per replicate): 4 groups were challenged intranasally with a nonlethal dose of H1N1 virus (1000 TCID_50_) (23) prior to treatment with AFB_1_ on d 1, d 7, and d 14 as described previously (24, 25), and the other two groups were given equivalent amounts of PBS intranasally. Among the 4 infected groups, three groups were given 10, 20, and 40 μg/kg b.w. AFB_1_ i.p. daily for 15 days, and the fourth group was given an equivalent amount of PBS i.p. Likewise, two uninfected groups were injected with equivalent amounts of PBS or AFB_1_ (40 μg/kg).

For the second randomized trial, WT mice were randomly divided into 2 groups (each group included 3 replicates, with 3 mice per replicate): the first group was given 3 mg/kg of TAK242 i.p.1 h prior to the other treatments, and the other group was given an equivalent amounts of PBS i.p.

For the third randomized trial, TLR4^−/−^ and WT mice were likewise divided into 2 groups (each group included 3 replicates, with 3 mice per replicate).

All mice from the second and third randomized trials were treated with equivalent amounts of H1N1 virus and 40 μg/kg of AFB_1_ as described for the first randomized trial.

### Histopathological examination and immunohistochemical staining

At the end of the experiments, mice were euthanized. Lung and spleen tissues were taken from each mouse. Approximately 75% of the lung tissue was stored at −80°C for the subsequent experiments, and the other 25% of the lung tissue was fixed in 4% formaldehyde for hematoxylin-eosin staining (H&E) according to standard protocols as described previously (26, 27) with some modifications. Briefly, lung tissue was fixed in 10-fold volume of 4% formaldehyde for 48 h. Next, samples were embedded in paraffin and cut into 4-μm-thick sections. One section from each tissue sample was stained with H&E.

For immunohistochemical staining, spleen tissues were incubated with a monoclonal antibody for TLR4 (Abcam, UK) and then incubated with an appropriate horseradish peroxidase (HRP)-conjugated secondary antibody. Subsequently, the HRP conjugates were visualized using a diaminobenzidine solution. Images were captured with a Pannoramic viewer (Pannoramic MIDI, 3D HISTECH), and data were analyzed using DensitoQuant software (QuantCenter, 3DHISTECH). A histochemistry score (H-score) was calculated according to a previously reported equation (28, 29).

### Fluorescent quantitative real-time PCR (qRT-PCR) analysis

Cells and lung and spleen tissues were collected to determine the relative mRNA expression levels of viral matrix (M) protein, TLRs, TNF-α and IL-10. Primers for the reference genes and target genes (Table 1) were designed and synthesized by Invitrogen based on known sequences. Briefly, total RNA was first extracted from tissues and cells using an RNAiso Plus kit (TaKaRa, Japan). First-strand cDNA was synthesized using a reverse transcription kit (TaKaRa, Japan). Subsequently, the samples of cDNA were subjected to qRT-PCR (TaKaRa, Japan) using specific primers with a no-cDNA template as a calibrator. The relative expression levels of the target genes were calculated by the 2^−ΔΔCT^ method with 18S or GAPDH as an endogenous reference gene.

**Table 1:** Primers sequences for real-time PCR.

**Source**	**Gene**	**Forward (5′-3′)**	**Reverse (3′-5′)**
Virus	*M*	GGGAAGAACACCGATCTTGA	CTCCGTTCCCATTAAGAGCA
Pig	*TLR1*	GAAACTACAAGGGCAGCTGG	GGGAAACTGAACACCTCCCT
	*TLR2*	AGACGCTGGAGGTGTTGG	AACGAAGCATCTGGGAGT
	*TLR3*	AAAACCAGCAACACGACT	TTGGAAAGCCCATAAAGA
	*TLR4*	AGAATGAGGACTGGGTGA	TGTAGTGAAGGCAGAGGT
	*TLR5*	GGCTCAACCAAACCAACG	GGGTGATGACGAGGAATAG
	*TLR6*	AACTCACCAGAGGTCCAA	TCTTCCCTGTCGATTCTC
	*TLR7*	GGCAAGTAGAGGACAT	GGTAGACCCTGAACAT
	*TLR8*	CGGCACCAGAAGAACG	GGCAGGTCAGGAGCAA
	*TLR9*	GGCCTTCAGCTTCACCTTGG	GGTCAGCGGCACAAACTGAG
	*TLR10*	ATGATTCGGCCTGGGTAAAG	TTGCCAGGATCAGAGTTTCC
	*IL-10*	CTGCCTCCCACTTTCTCTTG	TCAAAGGGGCTCCCTAGTTT
Mouse	*TNF-α*	GACTCAGATCATCGTCTC	GGAGTAGATGAGGTACAG
	*GAPDH*	CCACCCAGAAGACTGTGGAT	AAGCAGGGATGATGTTCTGG
	*TLR4*	CACTGTTCTTCTCCTGCCTGAC	CCTGGGGAAAAACTCTGGATA
	*18S*	TTGACGGAAGGGCACCACCAG	GCACCACCACCCACGGAATCG

*M, influenza A virus matrix protein; TLR, toll-like receptor; IL-10, interleukin 10; TNF-α, tumor necrosis factor-α; GAPDH, glyceraldehyde-phosphate dehydrogenase*.

### Western blot analysis

Cells and lung and spleen tissues were collected for western blotting analysis to assess the relative expression levels of viral nucleoprotein (NP), phosphorylated NFκB p65 (pp65) and TLR4. Briefly, total protein was extracted, and the protein concentration was measured with a BCA kit (Beyotime, China). The proteins were denatured, subjected to 10–15% of sodium dodecyl sulfate-polyacrylamide gel electrophoresis and then transferred to polyvinylidene difluoride membranes (Bio-Rad, USA). Next, the membranes were blocked for 2 h at room temperature (RT) in 5% bovine serum albumin (BSA) in Tris-buffered saline containing 0.1% Tween 20 (TBST), incubated overnight at 4°C with specific primary antibodies from (anti-NP, ab128193; anti-TLR4, ab13556; anti-pp65, ab76302 or anti-actin, ab14128; Abcam, UK), and then incubated for 1 h at RT with appropriate secondary antibodies (horseradish peroxidase-labeled anti-mouse or anti-rabbit secondary antibodies; Cell Signaling Technology, USA). Finally, the bound antibodies were visualized using an enhanced chemiluminescence kit (Beyotime, China).

### Determinations of TNF-α and IL-10 by ELISA

Whole blood from mice was collected from the retro-orbital plexus in heparinized tubes by a trained individual and was allowed to clot at RT. Sera were separated by centrifugation and stored at −80°C until analysis. The contents of TNF-α and IL-10 in sera were measured using ELISA kits (Jiancheng, China) according to the manufacturer's instructions.

### Short interfering RNA (siRNA) transfection

A pig TLR4-specific siRNA sequence, 5′-GGAUUUAUCCAGAUGUGAATT-3′, and a control siRNA sequence were obtained from a paper published by our coauthor (18). qRT-PCR was performed to determine the interfering efficiency of siTLR4. The siRNA experiment was carried out as our coauthor described previously (4). Briefly, PAMs were seeded in 12-well plates and were transfected for 6 h with X-tremeGENE siRNA transfection reagent (Roche, USA), siTLR4 and negative control siTLR4 diluted in medium according to the manufacturer's protocol, when the cells had reached approximately 70–80% confluence. Next, PAMs were infected with H1N1 virus and exposed to AFB_1_ for an additional 24 h for further experiments.

### Statistical analysis

Statistical analysis was conducted using Prism 6 (GraphPad Software, La Jolla, CA). Data are presented as the means ± SEM. Unpaired two-tailed Student's *t*-tests were performed to evaluate statistical significance for two-group comparisons, and ordinary one-way (nonparametric) ANOVA with Tukey's posttests and two-way ANOVA with Dunnett's posttests were performed to evaluate statistical significance for multigroup comparisons. A value of *P* < 0.05 was considered significant, and *P* < 0.01 was considered strongly significant.

## Results

### The cytotoxic effects of various concentrations of AFB_1_ on MDCK cells, A549 cells and PAMs

To remove the effects of AFB_1_-induced cytotoxicity on viral replication, the effects of various concentrations of AFB_1_ on cell viability were determined by MTT and LDH assays. As shown in Figures S1A–C, the viability of MDCK cells, A549 cells and PAMs decreased significantly when the AFB_1_ concentrations were greater than 0.5, 0.5, and 0.1 μg/ml, respectively. Correspondingly, LDH assay showed that LDH release increased markedly in MDCK cells, A549 cells, and PAMs when the AFB_1_ concentrations were greater than 0.5, 0.5, and 0.1 μg/ml, respectively (Figures S1D–F). Afterwards, DAPI staining was performed to determine the extent of apoptosis and thus to further assess the cytotoxicity of AFB_1_ on PAMs. As shown in Figure S1G, apoptosis began to occur when the AFB_1_ concentration reached 0.1 μg/ml and was identified by the condensation and fragmentation of nuclei. In addition, given that AFB_1_ was dissolved in DMSO, the effects of DMSO on MDCK cells, A549 cells and PAMs were also measured, and no significant differences were observed between the DMSO (1 μg/ml) group and either of the control groups (no DMSO and no AFB_1_). Taken together, these results suggest that AFB_1_ at concentrations between 0.01 and 0.25 μg/ml, 0.01 and 0.25 μg/ml, and 0.01 and 0.05 μg/ml are not toxic to MDCK cells, A549 cells and PAMs, respectively. Thus, for subsequent experiments, AFB_1_ was used at concentrations of 0.01, 0.05, and 0.25 μg/ml in both MDCK and A549 cells and at concentrations of 0.01, 0.025, and 0.05 μg/ml in PAMs.

### AFB_1_ promotes SIV replication in MDCK cells, A549 cells and PAMs

**Figure 1:**
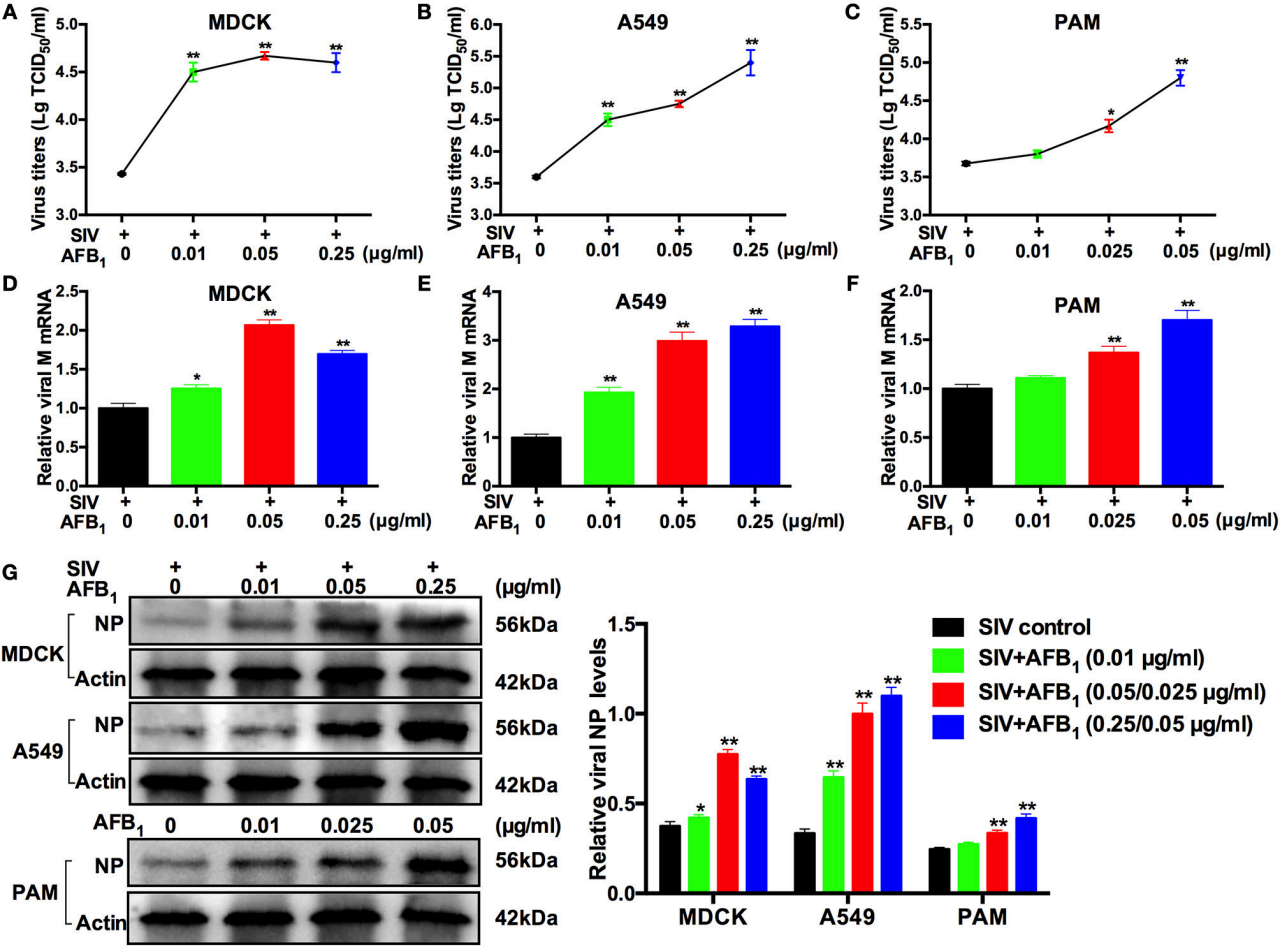
AFB_1_ promotes swine influenza virus (SIV) replication in multiple cell lines. Cells were infected with SIV (MOI = 1), and then incubated with various concentrations of AFB_1_ for 24 h. **(A–C)** Viral titers. Infectious virus particles were quantified by TCID_50_. **(D–F)** Viral M protein mRNA and **(G)** nucleoprotein (NP) levels were analyzed by qRT-PCR and western blotting, respectively. Data are presented as the means ± SEM of three independent experiments. Significance compared with the SIV control group, ^*^P < 0.05 and ^**^P < 0.01.

To investigate the potential role AFB_1_ plays in SIV replication, viral titers, viral M mRNA expression levels and NP expression levels were measured by TCID_50_, qRT-PCR and western blotting, respectively, as described previously (30). All cells were infected with SIV and then treated with various concentrations of AFB_1_ for 24 h. As shown in Figure 1, viral titers, M mRNA expression levels and NP levels were significantly increased in SIV-infected MDCK (Figures 1A,D,G) and A549 cells (Figures 1B,E,G) treated with 0.01–0.25 μg/ml AFB_1_ compared with levels in cells without AFB_1_ treatment. Correspondingly, viral titers, M mRNA expression levels and NP levels were also markedly increased in SIV-infected PAMs (Figures 1C,F,G) treated with 0.025–0.05 μg/ml AFB_1_ compared with levels in non-AFB_1_-treated PAMs. To confirm that the increase in SIV replication induced by AFB_1_ was not due to the presence of DMSO, we compared viral M mRNA expression of the three cell lines exposed to DMSO to that of the three cell lines exposed to medium and demonstrated that viral M mRNA expression in the three DMSO-exposed cell lines was identical to that in the cell lines exposed to medium alone (data not shown). Taken together, our results suggest that AFB_1_ exposure promotes SIV replication *in vitro*.

### AFB_1_ upregulates TLR4-NFκB signaling and promotes inflammatory responses in SIV-infected PAMs

**Figure 2:**
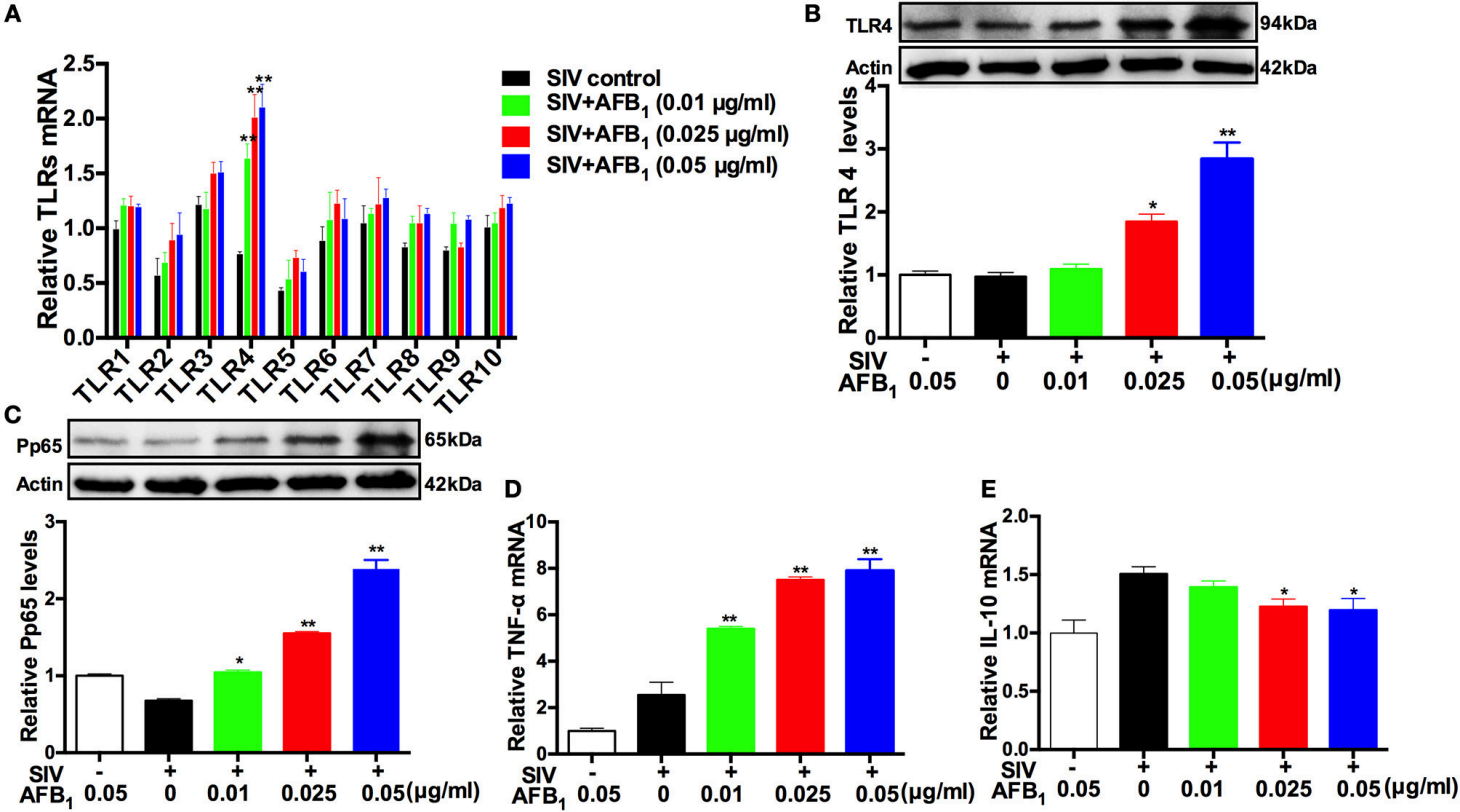
AFB_1_ upregulates TLR4-NFκB signaling and promotes inflammatory responses in SIV-infected PAMs. PAMs were incubated with or without SIV (MOI = 1), and then, the SIV-infected PAMs were incubated with various concentrations of AFB_1_ for 24 h. **(A)** Relative TLRs mRNA, **(B)** TLR4 protein, **(C)** phosphorylated NF-κB p65 (pp65), **(D)** TNF-α mRNA levels and **(E)** IL-10 mRNA levels. Data are presented as the means ± SEM of three independent experiments. Significance compared with the SIV control group, ^*^P < 0.05 and ^**^P < 0.01.

TLRs are a main family of pattern recognition receptors with a critical role in the activation of innate immune responses, but it has been proven that the overexpression or continuous activation of TLR4 can lead to excessive inflammatory responses or to injury in the body (16–18). To determine whether the promotion of SIV replication by AFB_1_ is associated with TLRs-induced innate immune responses or injury, the expression levels of TLRs 1-10 in SIV-infected PAMs were investigated. As shown in (Figure 2A), the relative expression of TLR4 mRNA was significantly elevated following exposure to 0.025–0.05 μg/ml AFB_1_ compared with the expression in the control group. This finding was confirmed by the marked increases in TLR4 protein levels (Figure 2B). TLR4 induces NFκB activation (15), and a previous study indicated that NFκB signaling involves pathogen- or cytokine-induced immune and inflammatory responses (31). To further confirm whether TLR4-NFκB was activated, the levels of pp65 were also determined. The results showed that 0.025–0.05 μg/ml AFB_1_ significantly increased the relative protein levels of pp65 (Figure 2C). The inflammatory response was quantified based on the expressions of the TNF-α and IL-10 genes, and the results indicated that 0.025–0.05 μg/ml AFB_1_ significantly increased the relative TNF-α mRNA level but decreased the relative IL-10 mRNA level (Figures 2D,E). Taken together, our results demonstrated that AFB_1_ upregulated TLR4-NFκB signaling and promoted inflammatory responses in the SIV-infected PAMs.

### TLR4 knockdown and BAY 11-7082 administration block the AFB_1_-promoted SIV replication and inflammatory responses in SIV-infected PAMs

**Figure 3:**
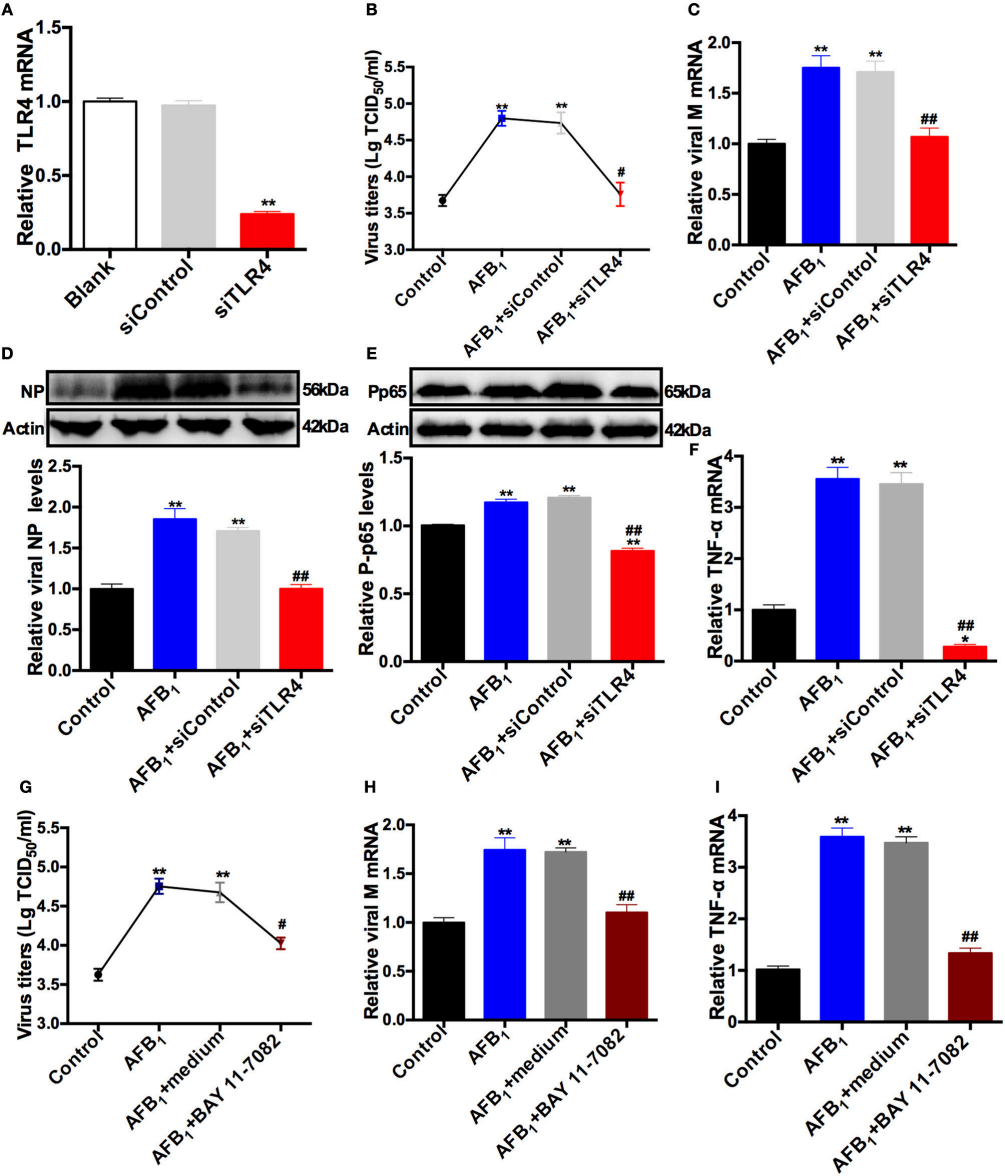
TLR4 knockdown and BAY 11-7082 reduce the AFB_1_-promoted SIV replication and inflammatory responses. PAMs were infected with SIV (MOI = 1) and then treated with (AFB_1_ group) or without (control group) 0.05 μg/ml AFB_1_. A TLR4-specific siRNA sequence was used to remove the effects of TLR4, and a control siRNA sequence was used as a negative control. **(A)** The knockdown efficiency of TLR4 siRNA in PAMs. **(B)** Viral titers and **(C)** relative viral M mRNA levels, **(D)** NP, **(E)** pp65 and **(F)** TNF-α mRNA levels. A specific inhibitor of NFκB, BAY 11-7082 (10 μM), was added to remove the effects of NFκB, and medium was used as a negative control. **(G)** Viral titers, **(H)** relative viral M mRNA levels, and **(I)** TNF-α mRNA levels. Data are presented as the means ± SEM of three independent experiments. Significance compared with the control group, ^*^P < 0.05, ^**^P < 0.01; significance compared with the negative control group, ^#^*P* < 0.05 and ^##^*P* < 0.01.

To further investigate the mechanism of SIV promotion by AFB_1_, a TLR4-specific siRNA sequence was used to remove the effects of TLR4, and a control siRNA sequence was used as a negative control. The interfering efficiency of siTLR4 was determined by qRT-PCR. As shown in (Figure 3A), TLR4 knockdown significantly decreased TLR4 mRNA expression by >70% compared with the expression in the blank; no significant difference in TLR4 mRNA expression was observed between the blank and siControl groups. In addition, our results demonstrated that 0.05 μg/ml AFB_1_ significantly elevated viral titers (Figure 3B), M mRNA expression (Figure 3C) and NP levels (Figure 3D) in SIV-infected PAMs compared to the corresponding parameters in control cells without AFB_1_. In contrast, TLR4 knockdown significantly reduced AFB_1_-promoted SIV replication, as indicated by lower viral titers, M mRNA expression and NP levels in the TLR4 knockdown group than in the siControl group; no significant difference in SIV replication was observed between the TLR4 knockdown and control groups (Figures 3B–D). These findings indicated that TLR4 knockdown blocked the promotion of SIV replication induced by AFB_1_. Likewise, TLR4 knockdown significantly reduced pp65 protein and TNF-α mRNA levels compared with the levels in the siControl group and even compared with the levels in the control group (Figures 3E,**F**), suggesting that TLR4 knockdown drastically counteracted the AFB_1_-promoted inflammatory responses in the SIV-infected PAMs.

Furthermore, our previous study indicated that BAY 11-7082 (10 μM), a specific inhibitor of NFκB, significantly reduces pp65 in PAMs and does not have cytotoxicity in PAMs (32). In the present study, BAY 11-7082 was used to further confirm the mechanism of SIV promotion by AFB_1_. The results showed that compared with medium alone, BAY 11-7082 significantly reduced the elevations in viral titers (Figure 3G), M mRNA levels (Figure 3H), and TNF-α mRNA levels (Figure 3I) promoted by AFB_1_ in SIV-infected PAMs, and no significant differences in the above parameters were observed between the BAY 11-7082 group and the control groups. Taken together, the results indicated that TLR4 knockdown and BAY 11-7082 blocked the AFB_1_-promoted SIV replication and inflammatory responses.

### AFB_1_ promotes SIV replication and lung damage induced by SIV in mice

**Figure 4:**
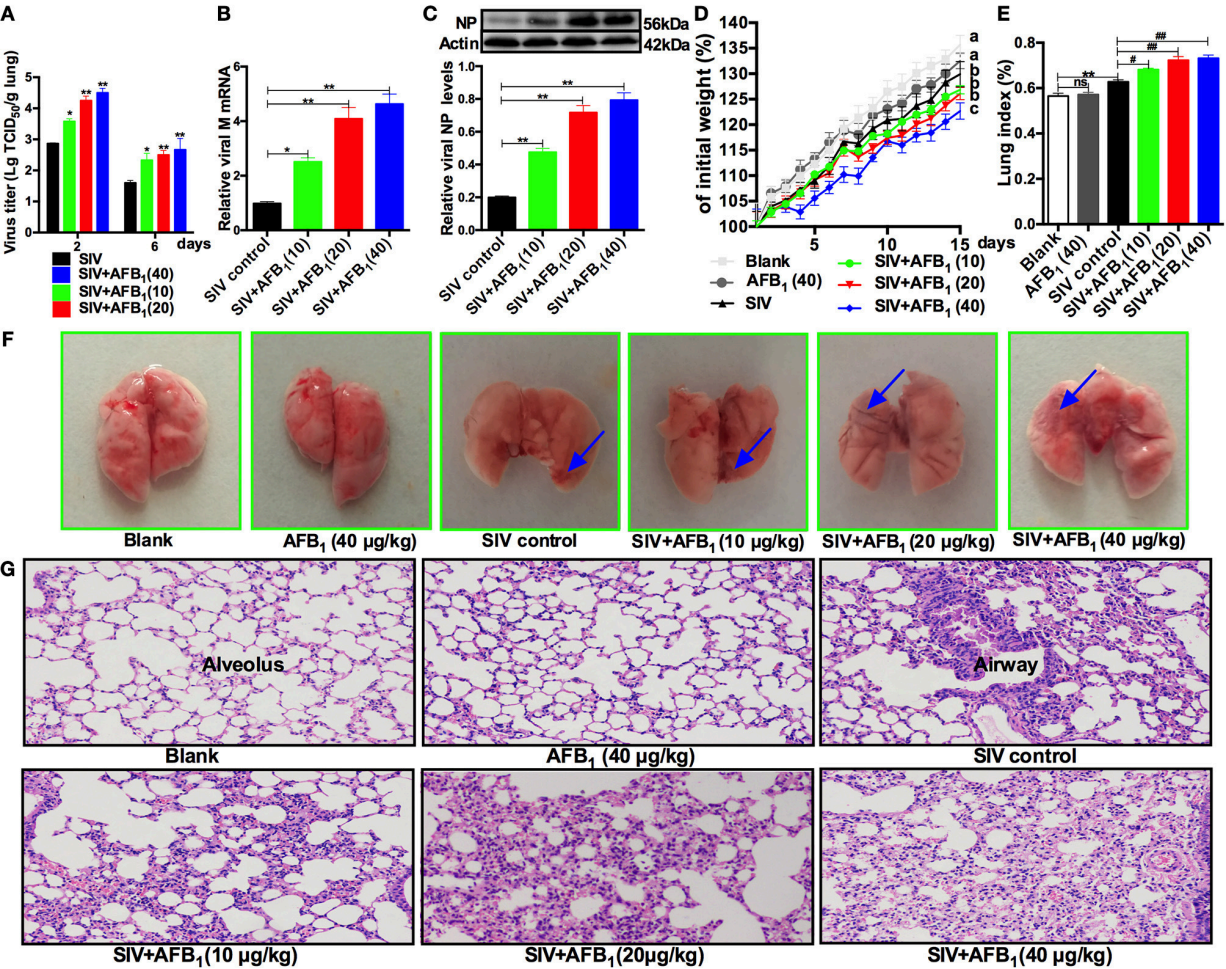
AFB_1_ promotes SIV replication and lung damage in mice. Anesthetized mice were infected intranasally with 1000 TCID_50_ of SIV or PBS on d 1, d 7, and d 14; injected intraperitoneally with various concentrations of AFB_1_ daily; and sacrificed at 15 days post infection (dpi). **(A)** Viral titers in the lung homogenates were determined by TCID_50_ on MDCK cells at 2 and 6 dpi. Data are shown as mean log_10_ TCID_50_ per gram of lung for three mice per group. The lung tissues were harvested at 15 dpi to assess viral replication as measured by **(B)** viral M mRNA and **(C)** NP levels. **(D)** Comparison of weight change expressed as a percentage of starting weight. **(E)** The lung index was calculated as the ratio of lung weight and body weight. **(F)** Representative images taken from nine mice in six groups as indicated. The areas of hemorrhage are denoted with the blue arrows. **(G)** Pathological changes in lungs. The mouse lungs were removed at 15 dpi, sectioned and stained with H&E for histological examination. Representative images from nine mice in each group were obtained at 200 × magnification. Data are presented as the means ± SEM of nine mice in each group; Different lowercase letters indicate significant differences (*P* < 0.05). ^*^^, #^*P* < 0.05, ^**^^, ##^*P* < 0.01, ns, not significant.

To further verify the *in vitro* results, lung tissues were taken from SIV-infected mice exposed to AFB_1_ to assess viral replication as indicated by viral titers (Figure 4A), M mRNA levels (Figure 4B) and NP levels (Figure 4C). As expected, AFB_1_ at doses of 10–40 μg/kg markedly increased viral titers, M mRNA levels and NP levels in lungs of SIV-infected mice compared with the levels in lungs of mice without AFB_1_. To further assess the impact of AFB_1_ on viral replication, weight gain (Figure 4D), the lung index (Figure 4E) and histological damage (Figures 4F,G) were determined. As expected, SIV-infected mice exhibited decreased weight gain, but enhanced the lung index and inflammatory cell infiltration compared with mice from the blank group, and these changes were aggravated following exposure to 10–40 μg/kg AFB_1_. In addition, 40 μg/kg AFB_1_ had no effects on these parameters in mice from the blank group (Figures 4D-G). Taken together, our data suggest that AFB_1_ promotes SIV replication and SIV-induced lung damage in mice.

### AFB_1_ promotes TLR4 expression and the inflammatory response in SIV-infected mice

**Figure 5:**
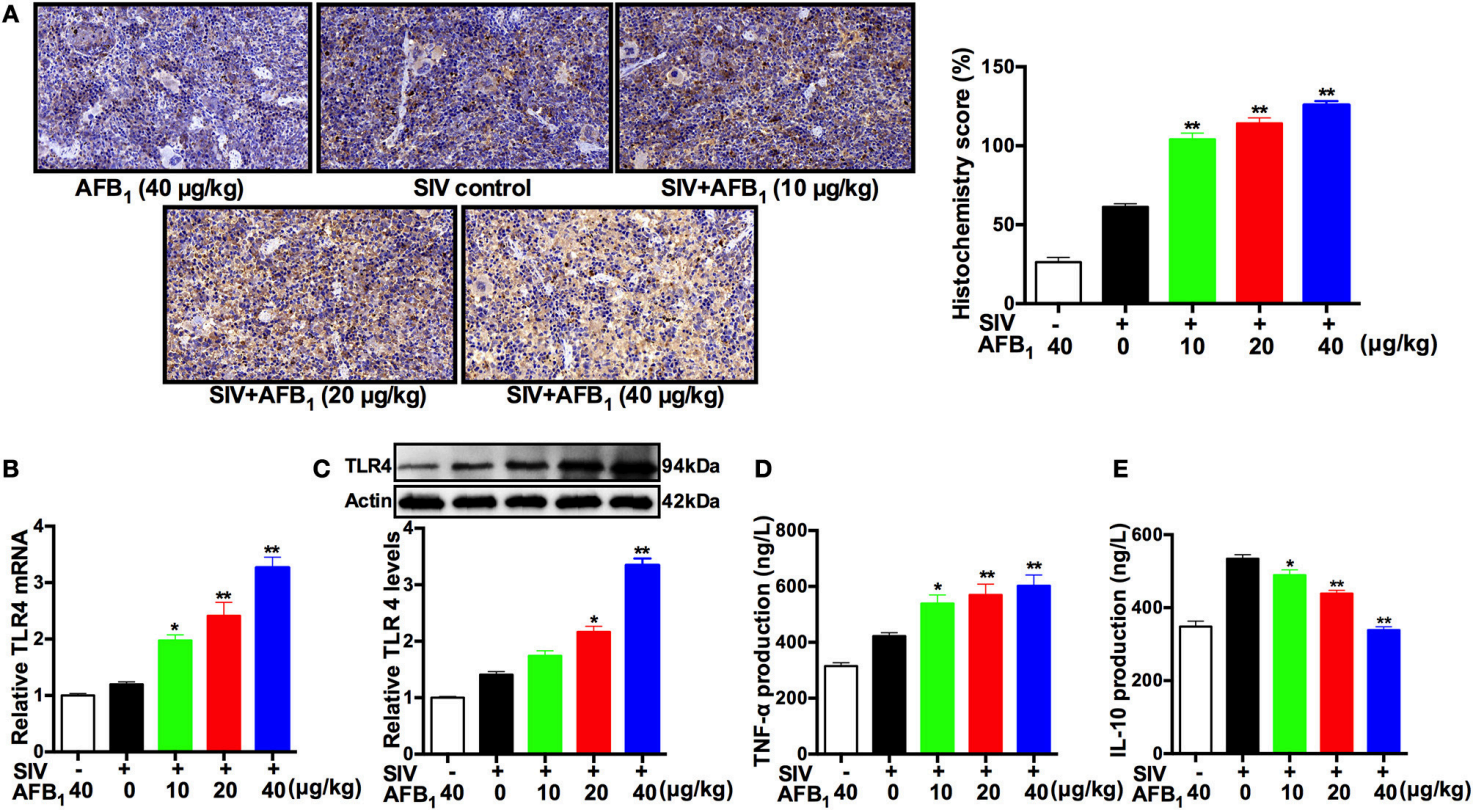
AFB_1_ promotes TLR4 expression and inflammatory responses in mice. Anesthetized mice were infected intranasally with 1000 TCID_50_ of SIV or PBS at d 1, d 7, and d 14; injected intraperitoneally with various concentrations of AFB_1_ daily; and sacrificed at 15 dpi. The spleen tissues were harvested at 15 dpi to assess TLR4 expression as measured by **(A)** TLR4 protein staining, **(B)** relative TLR4 mRNA levels and **(C)** TLR4 protein levels using immunohistochemistry, qRT-PCR and western blotting, respectively. **(D)** Serum TNF-α and **(E)** IL-10 levels. Representative immunohistochemistry images from nine mice in each group were obtained at 400 × magnification. Data are presented as the means ± SEM of three independent experiments. Significance compared with the SIV control group, ^*^P < 0.05 and ^**^P < 0.01.

To further verify the *in vitro* results, spleen tissues were taken from SIV-infected mice exposed to AFB_1_ to assess TLR4 expressions as indicated by TLR4 protein and mRNA levels. The immunohistochemical assay demonstrated that AFB_1_ at doses of 10–40 μg/kg significantly increased TLR4 expression (H-score) in the spleens of SIV-infected mice (Figure 5A). As expected, qRT-PCR and western blot assays supported the above results, demonstrating that AFB_1_ at doses of 10–40 μg/kg markedly increased TLR4 mRNA (Figure 5B) and protein levels (Figure 5C) in the SIV-infected mice compared with the levels in mice without AFB_1_. In addition, the inflammatory response was quantified by the release of TNF-α and IL-10, and the results showed that AFB_1_ at doses of 10 to 40 μg/kg markedly increased TNF-α release but significantly decreased IL-10 release in sera (Figures 5D,E). Taken together, our data suggest that AFB_1_ promotes TLR4 expression and the inflammatory response in SIV-infected mice.

### TAK242 and TLR4 knockout alleviates AFB_1_-promoted SIV replication, inflammation and lung damage in SIV-infected mice

**Figure 6:**
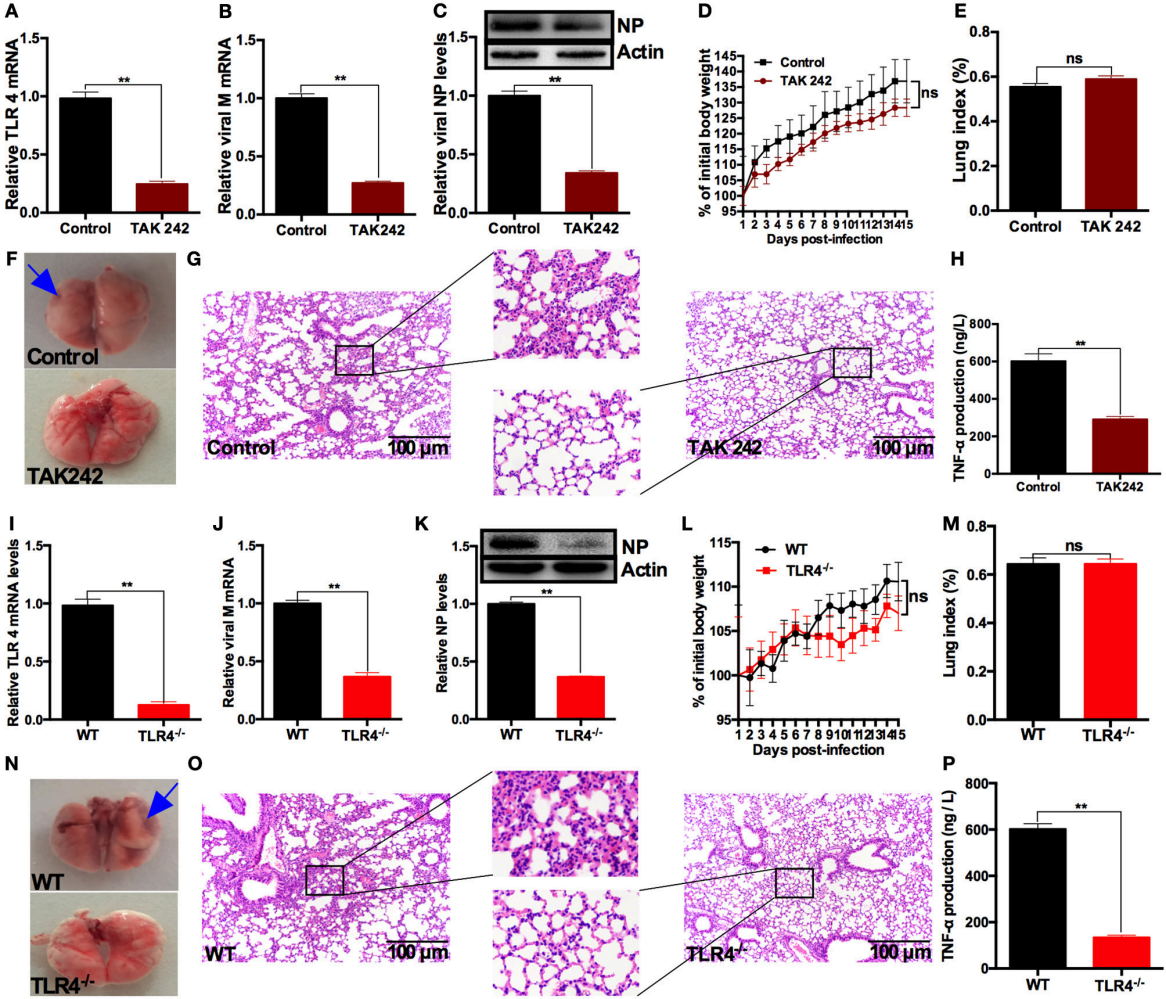
TLR4 deficiencies alleviate AFB_1_-promoted SIV replication, inflammation and lung damage in mice. Anesthetized mice were infected intranasally with 1000 TCID_50_ of SIV on d 1, d 7, and d 14 and were injected intraperitoneally with 40 μg/kg AFB_1_ daily. Mice from the TAK242 group were injected with 3 mg/kg TAK242 daily, and the mice from control group were given PBS. Mice were sacrificed at 15 dpi. **(A)** Relative TLR4 mRNA levels, **(B)** viral M mRNA levels, **(C)** viral NP levels, **(D)** body weight and **(E)** the lung index. **(F)** Representative images taken from mice in the control and TAK242 groups. The areas of hemorrhage are denoted with the blue arrows. **(G)** Pathological changes in lungs and **(H)** serum TNF-α in mice. TLR4^−/−^ and WT mice were infected intranasally with 1000 TCID_50_ of SIV on d 1, d 7, and d 14; injected intraperitoneally with 40 μg/kg AFB_1_ daily; and sacrificed at 15 dpi. **(I)** Relative TLR4 mRNA levels, **(J)** viral M mRNA levels, **(K)** viral NP levels, **(L)** body weight, and **(M)** the lung index. **(N)** Representative images taken from mice in the TLR4 knockout (TLR4^−/−^) and wild-type (WT) groups. The areas of hemorrhage are denoted with the blue arrows. **(O)** Pathological changes in lungs and **(P)** serum TNF-α in TLR4^−/−^ and WT mice. Data are presented as the means ± SEM. Significance compared with control/WT mice, ^**^P < 0.01; ns, not significant.

To determine the roles TLR4 plays in the promotion of SIV replication by AFB_1_ *in vivo*, the TLR4 inhibitor, TAK242, was used to treat mice (16). The results showed that TLR4 mRNA (Figure 6A), viral M mRNA (Figure 6B) and NP levels (Figure 6C) were markedly reduced in the presence of TAK242 compared with the levels in the no-TAK242 group, suggesting that TLR4 activation is required for the promotion of SIV replication by AFB_1_. However, no significant differences in weight gain (Figure 6D) or the lung index (Figure 6E) were observed between the TAK242 and control groups. Histological examination of lungs demonstrated that lung damage was alleviated after TAK242 administration (Figures 6F–G). In addition, TAK242 significantly reduced TNF-α content in sera (Figure 6H). Taken together, our results indicated that TAK242 alleviated AFB_1_-promoted SIV replication, inflammation and lung damage in SIV-infected mice.

To further confirm the roles TLR4 plays in the promotion of SIV replication by AFB_1_, TLR4^−/−^ mice were used in this study. The results showed that TLR4^−/−^ mice exhibited decreased TLR4 mRNA (Figure 6I), viral M mRNA (Figure 6J) and NP levels (Figure 6K) compared with WT mice, suggesting that TLR4 activation is indeed required for the promotion of SIV replication by AFB_1_. Likewise, no significant differences in weight gain (Figure 6L) and the lung index (Figure 6M) were observed between WT and TLR4^−/−^ mice. As expected, histological examination of lungs from TLR4^−/−^ mice did not reveal obvious lung damage (Figures 6N–O). In addition, the TNF-α content in sera of TLR4^−/−^ mice was lower than that in sera of WT mice (Figure 6P). Taken together, our results indicated that TLR4 knockout attenuated AFB_1_-promoted SIV replication, inflammation and lung damage in SIV-infected mice.

## Discussion

Swine are one of the species most sensitive to AFB_1_, and the maximum tolerance level of AFB_1_ for pigs is approximately 0.385 mg/kg of feed (33). On the contrary, mice are highly resistant to AFB_1_ (TD_50_ > 5,400 mg/kg b.w.) (34). According to the World Health Organization, in humans, AFB_1_ at concentrations of 30 to 50, 50 to 100, and 100 to 1,000 μg/kg b.w. produces mild, moderate and severe toxicity, respectively. According to the guidelines of the US Food and Drug Administration and the National Food Safety Standard (GB2761-2017, China), the maximum allowable dietary AFB_1_ concentrations for humans and animals are 20 and 300 μg/kg, respectively. However, it was previously unknown whether low-dose AFB_1_ could cause or exacerbate secondary diseases. Therefore, concentrations of 10, 20, and 40 μg/kg b.w. were used in this study. Our findings confirmed that 40 μg/kg AFB_1_ has no effects on the weight gain and lung function of mice, which is consistent with a previous study (34) and suggests that the promotion of SIV replication by AFB_1_ is not due to AFB_1_ toxicity. In addition, to remove the potential effects of AFB_1_-induced cytotoxicity on SIV replication, the safe concentrations of AFB_1_ were also determined by MTT and LDH assays and DAPI staining for further *in vitro* experiments.

Since the initial report in 1979 that AFB_1_ decreases interferon production by the influenza virus (35), few studies have been performed to determine its effects on SIV replication. Our study shows that AFB_1_ promotes SIV replication *in vivo* and *in vitro*. First, enhanced viral replication was observed in the MDCK cells, A549 cells and PAMs. Correspondingly, the *in vivo* results supported the conclusion of the *in vitro* experiments that AFB_1_ promotes SIV replication in mice. In addition, SIV-infected mice exposed to AFB_1_ also exhibited decreased weight gain but increased the lung index and lung damage. Our findings are consistent with the outcomes of SIV infection (36–38), suggesting that SIV infection is aggravated by AFB_1_.

Toll-like receptors (TLRs), which exist in porcine alveolar macrophages and in mice, are associated with the innate immune response (14, 39). Interestingly, viruses can evade the host immune response, thereby enhancing viral replication, when TLR4 is inhibited, but TLR4 antagonists can protect mice from lethal influenza infection (20). Therefore, the role of TLRs in the AFB_1_-induced promotion of viral replication was examined in our present study. Our data showed that AFB_1_ upregulated TLR4, but not other TLRs, in the SIV-infected PAMs. We investigated the underlying mechanism by using TLR4 knockdown and TLR4^−/−^ mice. The results showed that TLR4 knockdown and the inhibition of NFκB significantly reduced the AFB_1_-promoted SIV replication and inflammatory responses in PAMs, and TLR4 deficiencies also attenuated the AFB_1_-promoted SIV replication, inflammation and lung damage in mice. This may appear counterintuitive at first because the TLR4 pathway is often required for protection against influenza infection (40). Generally, TLR4 plays a critical role in the activation of innate immune responses to defend the body against pathogens. However, an increasing number of studies have shown that the overexpression and/or continuous activation of TLR4 can lead to excessive inflammatory responses or tissue damage in the body (16–18, 41). Our results are the first to suggest that AFB_1_ promotes SIV replication and SIV-related lung damage by activating the TLR4-NFκB pathway. This finding is supported by previous studies demonstrating that TLR4 antagonists or TLR4 knockout can prevent lethal influenza infection (20, 42). Therefore, we infer that AFB_1_ might promote TLR4 overexpression and excessive inflammatory responses and reduce tolerance (43), thereby promoting SIV replication.

Previous study indicated that the effects of proinflammatory cytokines were antagonized by anti-inflammatory cytokines such as IL-10 (43). In addition, a delicate balance between pro- and anti-inflammatory cytokine production is essential for the recovery from and defense against viral infection (44), which has roles in the maintenance of homeostasis and immunity. Accordingly, our data suggested that the inflammatory response was aggravated to defend against SIV infection, and IL-10 decreased and was not enough for the maintenance of homeostasis and immunity, thereby reducing the tolerance and increasing viral replication. On the contrary, excessive inflammatory responses can induce anti-inflammatory responses (19), and M2 macrophage polarization (anti-inflammatory macrophage phenotype) is TLR4 dependent (45). Therefore, it is likely that AFB_1_ promotes SIV replication via the TLR4-dependent induction of M2 macrophage polarization, but this possibility needs to be further studied.

In conclusion, our data suggest that AFB_1_ promotes SIV replication and SIV-induced lung damage by activating TLR4-NFκB signaling *in vitro* and *in vivo* or at least promotes these processes in a TLR4-dependent manner. This finding suggests a new risk of AFB_1_ exposure and reveals the vital role of TLR4-induced inflammation in the promotion of SIV replication and lung damage by AFB_1_, pointing to TLR4 as a potential therapeutic target for preventing lethal influenza infection.

## Author contributions

YS, FG, XC, and KH designed this project. YS, JS, ZL, and DL conducted the experiments. YS, KH, and DL wrote and revised the manuscript. FG, XC, KH, and DL gave helpful advice regarding the project. All authors reviewed the manuscript.

### Conflict of interest statement

The authors declare that the research was conducted in the absence of any commercial or financial relationships that could be construed as a potential conflict of interest.

## Supplementary material

Figure S1 Effects of various concentrations of AFB_1_ on cells. Cells were exposed to various concentrations of AFB_1_ for 24 h, and then subjected to **(A–C)** MTT, **(D–F)** LDH, and **(G)** DAPI staining assays for the detection of cell viability. A DMSO group was included to remove the effects of DMSO on cell viability, as the AFB_1_ was dissolved in DMSO. Cells without any AFB_1_ and DMSO were used as the control group. Cell nuclei were counterstained with DAPI to assess apoptosis, and the apoptotic cells were identified by the condensation and fragmentation of nuclei (yellow arrows). Data are presented as the means ± SEM of three independent experiments. Significance compared with the control group, ^*^*P* < 0.05 and ^**^*P* < 0.01.
