# Supplementary figures and images for: Aflatoxin B1 Promotes Influenza Replication and Increases Virus Related Lung Damage via Activation of TLR4 Signaling
Source: Front Immunol. 2018 Oct 4;9:2297. doi: 10.3389/fimmu.2018.02297 (PMC6180208; doi:10.3389/fimmu.2018.02297)

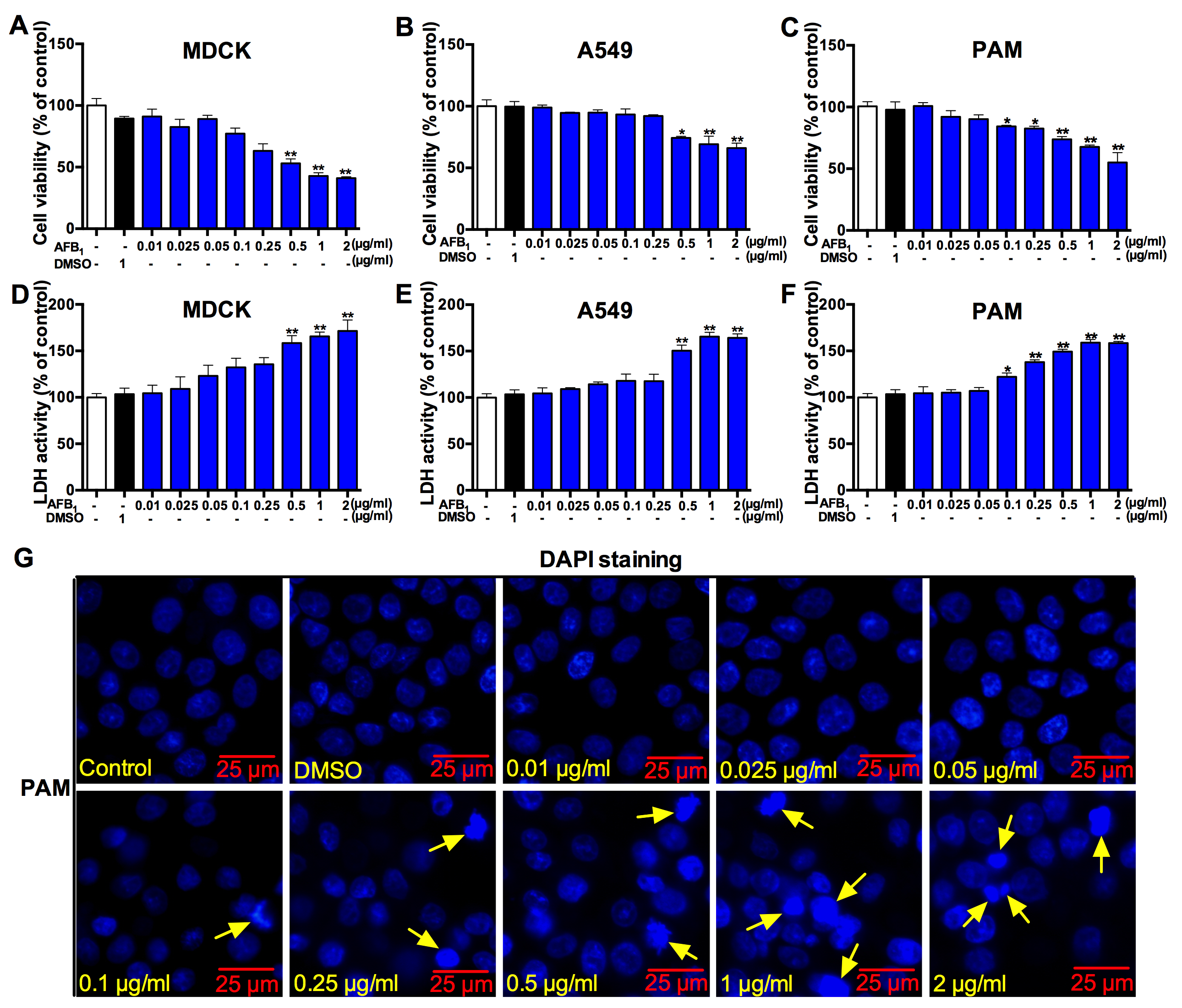

Supplement: Figure S1 — Effects of various concentrations of AFB1 on cells. Cells were exposed to various concentrations of AFB1 for 24 h, and then subjected to (A–C) MTT, (D–F) LDH, and (G) DAPI staining assays for the detection of cell viability. A DMSO group was included to remove the effects of DMSO on cell viability, as the AFB1 was dissolved in DMSO. Cells without any AFB1 and DMSO were used as the control group. Cell nuclei were counterstained with DAPI to assess apoptosis, and the apoptotic cells were identified by the condensation and fragmentation of nuclei (yellow arrows). Data are presented as the means ± SEM of three independent experiments. Significance compared with the control group, *P < 0.05 and **P < 0.01. [file Image_1.TIFF]
